# Prevalence of hyperuricemia and the population attributable fraction of modifiable risk factors: Evidence from a general population cohort in China

**DOI:** 10.3389/fpubh.2022.936717

**Published:** 2022-07-28

**Authors:** Huijing He, Pei Guo, Jiangshan He, Jingbo Zhang, Yujie Niu, Shuo Chen, Fenghua Guo, Feng Liu, Rong Zhang, Qiang Li, Shitao Ma, Binbin Zhang, Li Pan, Guangliang Shan, Minying Zhang

**Affiliations:** ^1^Department of Epidemiology and Statistics, Institute of Basic Medical Sciences, Chinese Academy of Medical Sciences, Beijing, China; ^2^School of Medicine, Nankai University, Tianjin, China; ^3^Beijing Physical Examination Center, Beijing, China; ^4^Hebei Key Laboratory of Environment and Human Health, Shijiazhuang, China; ^5^Department of Occupational Health and Environmental Health, Hebei Medical University, Shijiazhuang, China

**Keywords:** serum uric acid, body mass index, alcohol consumption, cigarette smoking, sedentary behavior, attributable fraction

## Abstract

Data on updated hyperuricemia prevalence in Beijing-Tianjin-Hebei (BTH) region in China, which is one of the world-class urban agglomerations, is sparse. Overweight/obesity, alcohol consumption, smoking and sedentary behavior are modifiable risk factors (MRFs) for elevated serum uric acid (SUA), but their population attributable fractions (PAFs) for hyperuricemia is still unclear. Using baseline data from the BTH Physical Examination General Population Cohort, we calculated the crude- and adjusted-prevalence of hyperuricemia based on the 30,158 participants aged 18–80 years. Hyperuricemia was defined as SUA >420 μmol/L in men and >360 μmol/L in women, or currently use of uric acid lowering drugs. Overweight/obesity, alcohol consumption, smoking and sedentary behavior were considered as MRFs and their adjusted PAFs were estimated. The prevalence of hyperuricemia was 19.37%, 27.72% in men and 10.69% in women. The PAFs and 95% confidence intervals for overweight, obesity were 16.25% (14.26–18.25%) and 12.08% (11.40–12.77%) in men, 13.95% (12.31–15.59%) and 6.35% (5.97–6.74%) in women, respectively. Alcohol consumption can explain 4.64% (2.72–6.56%) hyperuricemia cases in men, but with no statistical significance in women. Cigarette smoking contributed to 3.15% (1.09–5.21%) cases in men, but a much lower fraction in women (0.85%, 0.49–1.22%). Compared with sedentary time <2 h per day, the PAFs of 2–4 h, 4–6 h, and more than 6 h per day were 3.14% (1.34–4.93%), 6.72% (4.44–8.99%) and 8.04% (4.95–11.13%) in men, respectively. Sedentary time was not found to be associated with hyperuricemia in women. These findings concluded that hyperuricemia is prevalent in this representative Chinese adult general population with substantial sex difference. Four MRFs (overweight/obesity, alcohol consumption, cigarette smoking and sedentary behavior) accounted for a notable proportion of hyperuricemia cases. The PAF estimations enable the exploration of the expected proportion of hyperuricemia cases that could be prevented if the MRFs were removed, which warrants the public health significance of life-style intervention.

## Introduction

Serum uric acid (SUA) is the catabolic product of exogenous dietetic compounds and endogenous purines (1). Hyperuricemia is defined as the presence of elevated SUA. Hyperuricemia is not only necessary for the development of gout, but is also associated with cardiometabolic disease and kidney disorders (2–4), and even premature mortality (5–7). The prevalence of hyperuricemia is high in China. Results from the China National Health Survey revealed that the prevalence of hyperuricemia in mainland China is around 20% (8). Modifiable risk factors (MRFs), such as overweight/obesity, alcohol consumption, and sedentary behavior, were found to significantly increase the SUA level (8–11) and some major chronic diseases, such as hypertension, chronic kidney disease and dyslipidemia, which are commonly comorbid with hyperuricemia (12–14). Therefore, identifying individuals with high risk of hyperuricemia would benefit disease prevention and health promotion, and help the health professionals determine the priority population for intervention.

In epidemiological studies, relative risk (or odds ratio in a case-control scenario) is often used to assess the strength of health effect of a given exposure on a particular event. However, this measure did not consider prevalence of the exposure and thus cannot estimate the disease burden comprehensively (7). Population attributable fraction (PAF) is the proportion of cases for an outcome within a population that can be attributed to a certain risk factor, related with both exposure prevalence and its effect size (15). The focus on modifiable risk factors (MRFs), such as overweight/obesity, smoking, alcohol consumption and sedentary behavior, could provide real-world evidence on to what level the health hazards would have been avoided if people in high-risk changed their behavior. Furthermore, by calculating PAF, the importance of different MRFs could be estimated in sub-populations, thus helps policy makers to develop more tailored and targeted health promotion strategies. Previous cross-sectional studies have estimated several MRFs' PAF (8, 15, 16), but updated data derived from general adult Chinese is sparse.

The Beijing-Tianjin-Hebei (BTH) area is one of the world-class urban agglomerations in China. As the political center and third largest economy in China, this region accounts for 8.1% of China's population and is experiencing rapid socio-economic development and urbanization (17). Initiated in 2017, supported by the National Key R&D Program of China, the BTH Physical Examination Cohort aimed to explore determinants of non-communicable diseases using multidisciplinary methods, and update the real-world data to enhance health promotion. In recent years, the disease burden of hyperuricemia is increasing with various risk factors contributing to its rapid growth (18). Hence, using baseline data from the BTH Physical Examination Cohort, we aim to explore the hyperuricemia prevalence and the PAFs of MRFs in this general population.

## Methods

### Data resource and study population

This study was conducted using baseline data of the BTH Physical Examination General Population Cohort, which is a prospective cohort study initiated in 2017, containing over 30,000 adults recruited in the BTH area. The baseline survey was conducted from 2017 to 2018, with a multi-stage stratified cluster sampling method used in enrolling attendees at physical examination centers. The selection and sampling procedure: (1) among records of more than 200 physical examination centers in tertiary care hospitals, we randomly selected two or three medical examination centers by systematic sampling; (2) stable employees in organizations, institutions, and companies were selected with multiple labor categories (white collar, pink collar, or blue collar) from each selected physical examination center in the first stage. Finally, 100–200 registered organizations, institutions and companies for each occupation category were selected, within which individuals aged 18 years and above, living in the local place for at least 1 year, were asked to voluntarily participate in the study. Individuals who were pregnant, with severe mental or physical disorders were excluded.

The study has been carried out in accordance with the Declaration of Helsinki. Ethical approval was obtained from the ethics review boards of Nankai University (NKUIRB2016063), Tianjin First Central Hospital (2017N052KY), Tianjin Union medical center (2018C02) and Hebei Medical University (2016021). Written informed consent was obtained from each participant before the survey.

### Measurements

An id card reader has been used to automatically collect the subject's unique ID number, sex, birthday, and living address, and save the information into electronic database.

Sociodemographic and lifestyle information (age, sex, educational level, smoking, alcohol drinking, and daily sedentary time) were collected by standardized questionnaire interview. Immediately after the interview, a scanning software which was designed for this study was used to transform the hand-written information into electronic database in real time and save as excel files.

All subjects had standard physical and anthropometry measurements and provided blood samples for biochemical tests. This information will be linked with their annual physical examination electronic records to facilitate the follow-up.

During the anthropometric measurements, participants wore light clothing and were barefoot. Height (cm) and weight (kg) were measured using the same device (GL-310, Seoul, Korea) in different areas, with the accuracy on a decimal level.

Venous blood samples were drawn after an at least 8 h-overnight fast and were immediately sent to the laboratory of the physical examination centers for biochemical tests, which were performed using the same instrument (Hitachi 7600, Japan). SUA was measured by oxidization with the specific enzyme uricase.

### Outcome definition

Hyperuricemia was defined as SUA higher than 360 μmol/L (~6 mg/dl) in women and 420 μmol/L (~7.0 mg/dl) in men (12), or currently use of uric acid lowering drugs.

### Assessment of modifiable risk factors and other covariates

Overweight and obesity, ever-smoking, current alcohol drinking and daily sedentary time were considered as MRFs in the study. Underweight was defined as BMI < 18.5 kg/m^2^, normal weight was BMI between 18.5 kg/m^2^ and <24 kg/m^2^, overweight was BMI ≥ 24 kg/m^2^ but <28 kg/m^2^, and obesity was BMI ≥ 28 kg/m^2^, according to the recommendation suitable for the Chinese population (19). Information on smoking, alcohol drinking and daily sedentary time were collected by self-report in the face-to-face questionnaire interview. Participants who reported smoking for over half 1 year were defined as current smokers. Those who ever smoked but quit for at least half 1 year were defined as prior smokers. Individuals who consumed alcohol at least once per week were defined as current drinkers, and who had quit drinking alcohol for at least half 1 year were prior drinkers. Smoking status was grouped as never, prior, and current smoking. Ever-smoking was considered as either prior or current smoking. Alcohol drinking was categorized as never, prior and current drink. Daily sedentary time was classified as four groups: <2 h per day, 2–4 h per day, 4–6 h per day, and more than 6 h per day.

Estimated glomerular filtration rate (eGFR, ml/min per 1.73 m^2^) was calculated according to the Modification of Diet in Renal Disease equation for Chinese (c-MDRD) (20). Menopausal status was self-reported by female subjects in the face-to-face interview. Participants included in the survey were asked whether they had at least one menstrual bleeding in the past year to define menopause, excluding those caused by medications, hormones, medical conditions, or surgical procedures.

### Statistical analyses

The original data contains 31,311 participants. A total of 30,158 subjects were included in the final analyses after excluding subjects aged over 80 (*n* = 541, to avoid “healthy worker” effect), or with missing values on both serum uric acid level and information on uric-acid-lowering medication use (*n* = 436) or had an eGFR < 60 ml/min per 1.73 m^2^ (*n* = 176) to exclude possible kidney disorders (21).

The difference in basic characteristics between men and women were tested using chi-square test for grouped data or Wilcoxon rank test for non-normally distributed continuous data. The prevalence of hyperuricemia was adjusted using logistic regression models when compared among groups (22). General linear regression models (GLMs) were used to examine the relationship between MRFs and SUA. As odds ratio (OR) may overestimate the health effect when the outcome is not rare (10), prevalence ratio (PR) was used to estimate PAF. PAF was calculated as follow:


$$\begin{array}{l} PAF=\sum_{i=0}^{k}pd_{i}\left(\frac{PR_{i}-1}{PR_{i}}\right)=1-\sum_{i=\;0}^{k}\frac{pd_{i}}{PR_{i}} \end{array}$$

Pd_i_ refers to the proportion of cases in the i_th_ exposure level, and PR represents the prevalence risk (which is a simulation of relative risk) comparing the i_th_ exposure level with the unexposed group (*I* = 0) (23). A modified Poisson regression models were used to estimate the adjusted PAFs, which can be added to yield the integrated fraction that can be attributable to modifiable risk factors for hyperuricemia (24).

Furthermore, we did sensitivity analyses in three ways: (1) stratified analysis by menopause status to explore whether menopause modified the effect of MRFs on hyperuricemia; (2) excluding subjects with diagnosed chronic diseases to avoid possible inverse causality due to behavior change after awareness of disease; and (3) age- and sex- stratified analyses on PAFs estimation to learn whether there was age-varied effect of the MRFs on hyperuricemia.

For all coefficients and PR estimates, we calculated their 95% confidence intervals (CIs). A *p*-value (two-sided) < 0.05 was considered as statistically significant. SAS (version 9.4, SAS institute, Cary, NC) was used for statistical analyses.

## Results

### Basic characteristics and hyperuricemia prevalence

The mean age of the study population was 45.33 years (SD = 13.78 years), 45.99 years (SD = 13.79 years) in men and 44.65 years (SD = 13.74 years) in women. More than half (51%) were men. The median SUA level was 319 μmol/L (interquartile range: 121), 368.30 μmol/L (interquartile range: 107.11) and 271.18 μmol/L (interquartile range: 82.06) in men and women, respectively. The overall prevalence of hyperuricemia was 19.37%, 27.72% in men and 10.69% in women, respectively. The hyperuricemia prevalence in sub-groups, stratified by sex, was summarized in Table 1. Either in men or women, people in Tianjin area had lower hyperuricemia prevalence (24.24% in men and 8.98% in women). The patterns of hyperuricemia prevalence with aging were different between sexes, decreasing in men but increasing in women (both *P* for trend < 0.001). People with higher BMI, ever smoking cigarettes, ever drinking alcohol, with longer daily sedentary time had higher prevalence of hyperuricemia (Table 1).

**Table 1:** The prevalence of hyperuricemia in general population from the Beijing, Tianjin, and Hebei areas (*n* = 30,158).

	**Male (*****n*** = **15,381)**					**Female (*****n*** = **14,777)**				
	***N***	**Crude-** ***P***	**Adjusted-** ***P*** ^*^ **and 95% CI**			***N***	**Crude-** ***P***	**Adjusted-** ***P*** ^*^ **and 95% CI**		
**Demographic characteristics**										
**Areas**										
Beijing	4,854	34.84	33.15	31.81	34.52	3,709	12.91	13.16	12.09	14.31
Tianjin	4,149	24.24	23.47	22.43	24.55	7,450	8.98	9.09	8.44	9.77
Hebei	6,378	24.73	25.02	23.70	26.39	3,618	11.94	11.92	10.89	13.04
**Age groups**										
20–	2,062	32.88	32.62	30.62	34.70	2,290	9.56	10.04	8.84	11.38
30–	4,014	35.58	35.31	33.81	36.83	4,179	8.69	9.13	8.27	10.08
40–	3,336	27.52	27.57	26.07	29.12	3,444	7.61	7.86	6.99	8.82
50–	3,313	22.64	22.85	21.44	24.32	2,383	13.85	14.55	13.15	16.07
60–80	2,656	18.41	18.94	17.47	20.50	2,481	16.36	16.90	15.45	18.45
**Ethnicity**										
Han	14,844	27.53	26.92	26.17	27.69	14,141	10.69	11.25	10.71	11.82
Others	537	32.77	29.79	26.08	33.79	636	10.85	11.78	9.39	14.68
**Educational level**										
Primary/junior high school	1,805	19.56	20.52	18.62	22.57	1,166	11.49	10.32	8.70	12.21
Senior high school	2,257	23.97	24.75	22.95	26.64	1,596	14.41	12.32	10.80	14.01
College	9,003	29.98	29.13	28.16	30.13	9,036	10.98	11.76	11.06	12.48
Postgraduate	670	28.93	26.66	24.79	28.62	224	7.52	9.29	8.14	10.59
**Modifiable risk factors**										
**Body mass index (kg/m^2^)**										
<24	5,075	18.03	17.15	16.13	18.22	8,954	6.85	7.39	6.83	7.99
24-	6,707	27.82	28.00	26.89	29.14	4,078	14.71	15.05	13.93	16.25
≥28	3,260	41.96	41.44	39.69	43.21	1,328	23.64	23.73	21.45	26.16
**Cigarette smoking**										
Never	8,941	27.14	25.50	24.56	26.46	14,470	10.59	11.18	10.64	11.74
Prior	1,197	27.23	32.06	29.27	34.99	55	16.36	16.58	8.81	29.02
Current	5,243	28.80	28.40	27.15	29.68	252	15.48	15.70	11.64	20.84
**Alcohol drinking**										
Never	8,671	25.64	25.01	24.08	25.98	13,998	10.66	11.25	10.69	11.82
Prior	407	24.82	29.00	24.52	33.93	48	14.58	14.68	7.12	27.85
Current drinking	6,303	30.76	29.65	28.49	30.84	731	11.08	11.52	9.34	14.12
**Daily sedentary time**										
<2 h	1,685	22.20	20.96	19.06	22.99	1,305	11.11	9.80	8.33	11.48
2–4 h	3,130	27.41	26.39	24.85	27.99	2,605	11.36	10.43	9.32	11.66
4–6 h	4,548	29.64	28.95	27.61	30.33	4,169	10.70	11.33	10.36	12.38
>6 h	6,018	27.97	27.90	26.68	29.15	6,698	10.35	12.06	11.20	12.98

* *The adjusted covariates were age and areas*.

*CI, confidence interval*.

### Distribution of modifiable risk factors and their effect on SUA

The distribution of MRFs in different age groups and sex were presented in Table 2. The proportions of overweight and obesity in men were much higher than that in women in each age group. Over 60% of men aged 30 and above were overweight. An increasing trend of overweight and obesity with aging was observed in women (*P* for trend < 0.001). The proportions of other MRFs except for long sedentary time were also higher in men than in women (Table 2).

**Table 2:** The distribution of modifiable risk factors in the study population (*n* = 30,158).

**Age-groups**	**20–**		**30–**		**40–**		**50–**		**60–80**		**Overall**		***P***
	***n***	**%**	***n***	**%**	***n***	**%**	***n***	**%**	***n***	**%**	***n***	**%**	
**Men**													
Body mass index categories													<0.001
Under/normal weight	915	44.37	1,298	32.34	1,037	31.09	1,004	30.30	821	30.91	5,075	33.00	
Overweight	716	34.72	1,673	41.68	1,473	44.15	1,577	47.60	1,268	47.74	6,707	43.61	
Obesity	406	19.69	941	23.44	746	22.36	653	19.71	514	19.35	3,260	21.19	
Cigarette smoking													<0.001
Never	1,368	66.34	2,603	64.85	1,906	57.13	1,602	48.35	1,462	55.05	8,941	58.13	
Ever	54	2.62	130	3.24	242	7.25	291	8.78	480	18.07	1,197	7.78	
Current	640	31.04	1,281	31.91	1,188	35.61	1,420	42.86	714	26.88	5,243	34.09	
Alcohol drinking													<0.001
Never	1,313	63.68	2,345	58.42	1,736	52.04	1,661	50.14	1,616	60.84	8,671	56.37	
prior	18	0.87	36	0.90	86	2.58	121	3.65	146	5.50	407	2.65	
Current	731	35.45	1,633	40.68	1,514	45.38	1,531	46.21	894	33.66	6,303	40.98	
Daily sedentary time													<0.001
<2 h	179	8.68	269	6.7	370	11.09	531	16.03	336	12.65	1,685	10.96	
2–4 h	408	19.79	682	16.99	645	19.33	656	19.80	739	27.82	3,130	20.35	
4–6 h	566	27.45	1,252	31.19	1,025	30.73	925	27.92	780	29.37	4,548	29.57	
>6 h	909	44.08	1,811	45.12	1,296	38.85	1,201	36.25	801	30.16	6,018	39.13	
**Women**													
Body mass index categories													<0.001
Under/normal weight	1,824	79.65	2,882	68.96	1,968	57.14	1,201	50.40	1,079	43.49	8,954	60.59	
Overweight	298	13.01	867	20.75	1,054	30.60	868	36.42	991	39.94	4,078	27.60	
Obesity	88	3.84	281	6.72	323	9.38	272	11.41	364	14.67	1,328	8.99	
Cigarette smoking													<0.001
Never	2,254	98.43	4,095	97.99	3,391	98.46	2,328	97.69	2,402	96.82	14,470	97.92	
Ever	36	1.57	84	2.01	53	1.54	55	2.31	79	3.18	307	2.08	
Alcohol drinking													<0.001
Never/prior	2,124	92.75	3,983	95.31	3,272	95.01	2,266	95.09	2,401	96.78	14,046	95.05	
Current	166	7.25	196	4.69	172	4.99	117	4.91	80	3.22	731	4.95	
Daily sedentary time													<0.001
<2,h	147	6.42	248	5.93	335	9.73	232	9.74	343	13.83	1,305	8.83	
2–4 h	366	15.98	498	11.92	567	16.46	463	19.43	711	28.66	2,605	17.63	
4–6 h	622	27.16	1,144	27.37	987	28.66	723	30.34	693	27.93	4,169	28.21	
>6 h	1,155	50.44	2,289	54.77	1,555	45.15	965	40.50	734	29.58	6,698	45.33	

SUA was significantly influenced by the MRFs, especially in men (Table 3). Excess body weight increased the SUA in both sexes. An average of 50.82 (95%CI: 47.30–54.35) μmol/L higher SUA level was observed in obese men, compared with under/normal weight men. Similar result was observed in obese women, with an elevated SUA of 50.04 (95%CI: 46.28–53.80) μmol/L, compared with their normal weight counterparts. Interestingly, longer daily sedentary time seemed to only influence male participants, without positive association observed in women. There were few women currently smoking or drinking alcohol, leading to marginal or non-significant statistical analysis results (Table 3).

**Table 3:** The effect of modifiable risk factors on serum uric acid, stratified by sex.

	**Men**				**Women**			
**Modifiable risk factors**	***B***	**95% CI**		***P***	***B***	**95% CI**		***P***
**BMI categories**										
Under/normal weight	Ref	NA		NA	Ref	NA		NA
Overweight	23.51	20.61	26.41	<0.001	25.86	23.46	28.25	<0.001
Obesity	50.82	47.30	54.35	<0.001	50.04	46.28	53.80	<0.001
**Cigarette smoking**										
Never	Ref	NA		NA	Ref	NA		NA
Prior	8.77	3.68	13.87	<0.001	13.07	−5.90	32.03	0.177
Current	2.39	−0.53	5.32	0.108	10.31	0.58	20.05	0.038
**Alcohol drinking**										
Never	Ref	NA		NA	Ref	NA		NA
Prior	3.98	−4.1532	12.11	<0.001	1.29	−17.41	19.98	0.893
Current	8.68	5.97	11.39	<0.001	2.85	−2.16	7.85	0.265
**Daily sedentary time**										
<2 h	Ref	NA		NA	Ref	NA		NA
2–4 h	7.19	2.45	11.93	0.003	−1.31	−5.60	2.98	0.550
4–6 h	12.01	7.34	16.69	<0.001	−0.67	−4.84	3.50	0.752
>6 h	11.27	6.63	15.91	<0.001	0.09	−3.98	4.15	0.966

*B, regression coefficient; CI, confidence interval; NA, not applicable; BMI, body mass index, kg/m^2^*.

*The covariates adjusted in the regression model were age, geographic areas, body mass index, alcohol consumption, smoking status, daily sedentary time and serum creatinine. Menopausal status was additionally adjusted in women*.

### The PAF of modifiable risk factors for hyperuricemia

The sex specific PR, PAF of MRFs for hyperuricemia were presented in Table 4. The overall PAF for excess body weight (overweight and obesity) in men was 28.31% (16.25% for overweight and 12.08% for obesity), indicating that 28.31% of the hyperuricemia cases in the male participants can be explained by excess body weight. In women, the PAF of excess body weight was 20.30% (13.95% for overweight and 6.35% for obesity). Consistent with the GLM results, sedentary behavior was only found to be associated with male hyperuricemia, men who had more than 6 sedentary h per day contributed to 8% of cases. Alcohol drinking and smoking had relatively low PAFs both in men and women (Table 4). After stratified by body weight, we found that men who had lower body weight were more likely to be affected by sedentary behavior (see in Supplementary Table S1).

**Table 4:** The prevalence ratio, population attributable fraction of modifiable risk factors for hyperuricemia, stratified by sex.

**Modifiable risk factors**	**% of exposure**	**PR**	**95% CI of PR**		**PAF (%)**	**95%CI or PAF**		***P***
**Male**										
Overweight	44.59	1.57	1.46	1.68	16.25	14.26	18.25	<0.001
Obesity	21.67	2.26	2.10	2.42	12.08	11.40	12.77	<0.001
Current drinking	40.98	1.13	1.07	1.19	4.64	2.72	6.56	<0.001
Ever smoking	41.87	1.08	1.02	1.14	3.15	1.09	5.21	0.003
**Daily sedentary time**										
2–4 h	20.35	1.18	1.06	1.31	3.14	1.34	4.93	<0.001
4–6 h	29.57	1.29	1.17	1.42	6.72	4.44	8.99	<0.001
>6 h	39.13	1.26	1.13	1.38	8.04	4.95	11.13	<0.001
**Female**										
Overweight	28.40	1.97	1.74	2.19	13.95	12.31	15.59	<0.001
Obesity	9.25	3.19	2.77	3.62	6.35	5.97	6.74	<0.001
Current drinking	4.95	0.84	0.63	1.04	−0.01	−0.02	0.005	0.195
Ever smoking	2.08	1.69	1.19	2.19	0.85	0.49	1.22	<0.001
**Daily sedentary time**										
2–4 h	17.63	1.06	0.86	1.26	1.00	−2.17	4.16	0.538
4–6 h	28.21	1.05	0.87	1.26	1.64	−3.23	6.51	0.509
>6 h	45.33	1.14	0.93	1.34	5.41	−1.73	12.55	0.137

*PR, prevalence ratio; PAF, population attributable fraction; CI, confidence interval*.

*The covariates adjusted in the regression model were age, geographic areas, body mass index, alcohol consumption, smoking status, daily sedentary time, and serum creatinine. Menopausal status was additionally adjusted in women*.

**Figure 1:**
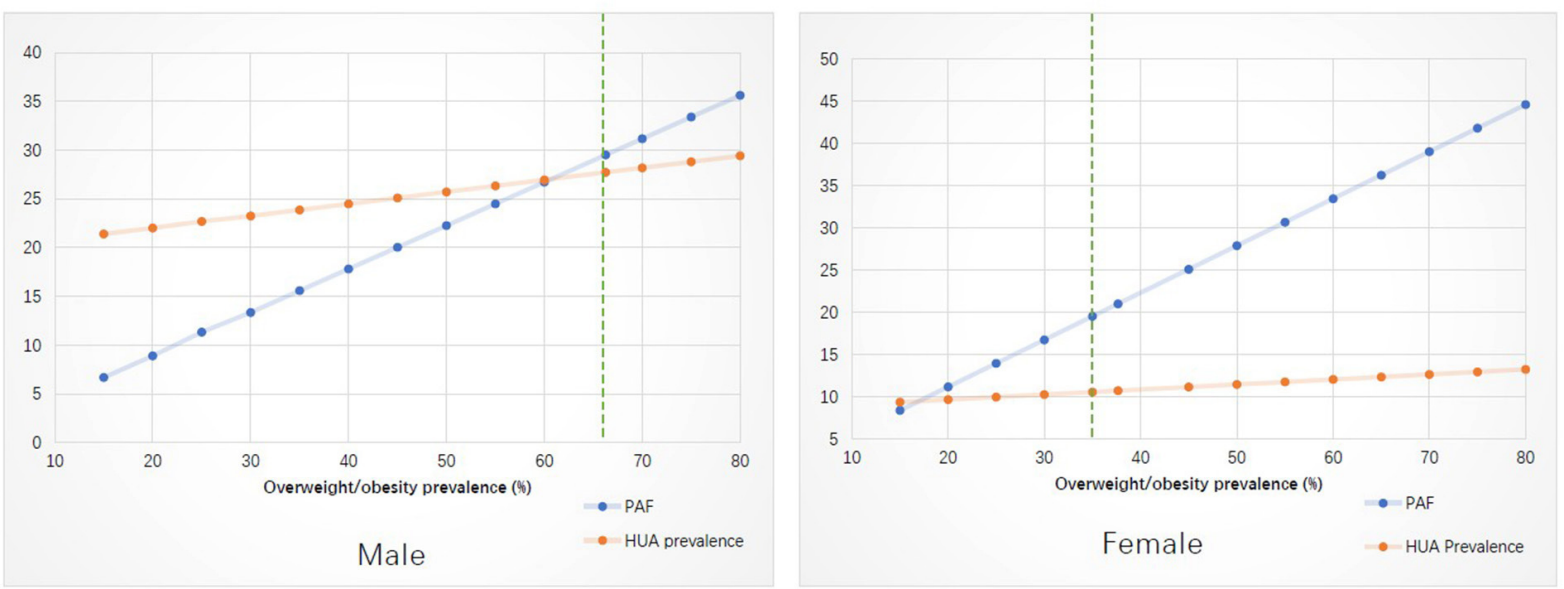
Stimulation analysis on the effect of changed modifiable risk factors on the prevalence of hyperuricemia. PAF, population attributable fraction; HUA, hyperuricemia.

In the simulation analysis (Figure 1), taking overweight/obesity for example, we demonstrated the change of hyperuricemia prevalence due to PAF variation. More details for Figure 1 were available in Supplementary Table S2.

### Sensitivity analyses

The results of sensitivity analyses were shown in Supplementary Tables S3–S5. After stratified by menopausal status, the main findings were consistent with results in Table 4. Post-menopausal women were more likely to be influenced by excess body weight (Supplementary Table S3). After excluding people suffering from chronic diseases, the PAFs estimates kept consistent with primary analyses (Supplementary Table S4). Middle-aged men and elder women had larger PAFs in the age-stratified analyses (Supplementary Table S5).

## Discussion

In this representative sample of general population of Chinese adults, we reported the most updated data of hyperuricemia prevalence in central China. Furthermore, we took overweight/obesity, alcohol drinking, cigarette smoking, and sedentary behavior as modifiable risk factors and estimated their populational attributable fractions for hypouricemia cases, thus highlights the life-style intervention priorities in the prospective of public health importance.

Our study revealed a high prevalence of hyperuricemia in BTH area of China. In line with previous studies, there is sex difference in hyperuricemia prevalence. In the Henan Rural Cohort study from 2015 to 2017, the crude hyperuricemia prevalence was 12.80% in men and 8.56% in women, respectively (11). Revealed by the China National Health Survey (2012–2017), the hyperuricemia prevalence was 25.07% in men and 15.94% in women (8). Using baseline data of the China Health and Retirement Longitudinal Study in 2010, Song et al. (25) reported a lower prevalence of hyperuricemia, 7.9 vs. 4.9% for men and women. A cross-sectional survey from 2013 to 2014 in southwest China among middle-aged adults also showed a higher hyperuricemia prevalence in men than in women (17.3 vs. 10.0%) (26). The inconsistence among prevalence may be attributed to the population heterogeneity, disparity on covariates distribution, survey time variation of hyperuricemia as its increasing trend, or geographic difference (8, 9, 27).

In contrary to women, the prevalence of hyperuricemia in men decreased with aging, with the highest prevalence in the younger group (over 30% in the 20–40 age group). The sex difference, therefore, is shrinking as aging, same with the results observed in previous observational study (28). The increasing prevalence among women may be influenced by both aging and menopausal status (29). Menopause has been suggested as a female-specific cardiovascular risk factor by the American Heart Association (30). Higher level of SUA has been observed in post-menopausal women in both European and Asian populations (31–33). Higher prevalence of hyperuricemia with aging in female requires more attention on aged women, especially post-menopausal individuals.

Life-style risk factors, such as excess adiposity, alcohol consumption, cigarette smoking, and sedentary behavior, were found to be associated with hyperuricemia (8, 9, 11, 34–38). Mendelian randomization studies have demonstrated the causal associations among excess adiposity and alcohol consumption with elevated SUA (16). Our study also revealed the positive effect of overweight/obesity in both sexes. Notably, men had a high overweight/obese prevalence, even in the youngest group. Since PAF incorporates both exposure proportion and health effect size, it is not surprising that excess body weight played a predominant role (28.33%) in the overall PAF of hyperuricemia. For women, although their exposure proportion was lower than men, given the greater effect size of exposure, the PAF of excess body weight in women was also substantial (20.3%). Few studies have explored the PAF of excess body weight. Data from the Third National Health and Nutrition Examination Survey (NHANES-III) revealed a 44% PAF of overweight/obesity for hyperuricemia (10); data from three UK cohorts reported 59.0–68.6% PAFs for BMI > 25 kg/m^2^ among non-gout population (16). Our previous study using data from CNHS showed a similar PAF of overweight/obesity to the present study (20.6 vs. 28.33% in men; 18.1 vs. 20.3% in women). The higher PAFs in the western population are possibly due to their high exposure prevalence or using OR instead of PR to estimate the effect size (which may lead to overestimation when the outcome is not rare) and difference in genetic background.

Previous studies reported a varied PAF of alcohol consumption from 8 to 21.6% (8, 15, 38). Our study revealed a minor effect of current alcohol drinking on hyperuricemia in men. Possible reasons for this inconsistence may be attributed to different consumption dose of alcohol, as there is a dose-response relationship between alcohol intake and SUA level (39), and the lower exposure proportion in the study population (especially in women, <5%). More details on alcohol intake is needed in the follow-up data collection to enable further analysis.

The associations between smoking and hyperuricemia are not consistent among studies (40). In some cross-sectional studies, inverse relationships were observed that current smoking had lower SUA (41, 42). In Han et al.'s study, only passive smoking was found to be associated with elevated SUA, but not for active smoking (37). Our study revealed that ever-smoking was associated with a higher likelihood of hyperuricemia. The possible reason is that ever-smoking maybe related to other health disorders, such as metabolic syndrome or other cardiometabolic conditions which were believed to be associated with hyperuricemia (43, 44). The sensitivity analyses excluding people with chronic disease proved this hypothesis to some extent: ever smoking lost its statistical significance in the association with hyperuricemia. Therefore, the explanation of the contribution of smoking to hyperuricemia should be with caution and further be understood in prospective study.

Our study revealed sex-difference of the effect of sedentary behavior on elevated SUA. Men with longer sedentary time had increased odds of hyperuricemia, about 18% of hyperuricemia cases in men could be attributed to longer sedentary time. Although physical activity has been proved to be able to reduce the increases in mortality risk from high SUA (45), the role of sedentary behavior has received limited attention. Park et al. observed a positive association between sedentary behavior and hyperuricemia among the Korean population (46). In a Chinese population, per hour increased sedentary time was associated with a 5% higher hyperuricemia risk (11). As sedentary behavior may lead to excess body weight (47), we further explore the effect of sedentary time in men stratified by body weight. Interestingly, men who had lower body weight were more likely to be affected by sedentary behavior. The possible reason could be the competing risk among MRFs. As excess adiposity had the strongest effect size among MRFs, the effect of alcohol consumption, smoking or sedentary behavior may be attenuated in obese population. Inconsistent with previous study (48), women, but not men, was observed to have higher risk of hyperuricemia due to sedentary behavior. Possible reasons for this inconsistency may be due to difference in study population and varied exposure levels of MRFs. Hong et al.'s study focused on a population in Yunnan province, Southeast China (48), where our previous research has demonstrated a low prevalence of overweight/obesity, but high prevalence of hyperuricemia (8). As above discussed, competing risk may exist in females where excess adiposity perhaps played predominant role in developing hyperuricemia in the study population.

The prevention strategies for high-risk subjects should be identified based on their personal health profiles. Revealed by our study, obese men currently drinking alcohol and ever smoking cigarettes with long sedentary time, maybe the key population for hyperuricemia prevention.

Our study had some strengths. First, using a representative sample with large sample size, we reported the updated hyperuricemia prevalence in the core area of China where is experiencing fast economic development and lifestyle changing. Second, several essential modifiable risk factors were considered in the health effect estimates. Most importantly, by calculating their PAFs, we can estimate the expected proportion of hyperuricemia cases that could be prevented if the MRFs were removed (23), and this is of great public health significance. Third, we for the first time estimate the PAF of sedentary time on hyperuricemia in a general population, which could provide new evidence for a rapid urbanization area where young adults may be influenced more by sedentary behavior. The limitations of our study should also be acknowledged. First, the baseline data of a cohort study cannot guarantee the causal relationship between MRFs on hyperuricemia, longitudinal observation will be needed when enough follow-up data is available. Second, we did not collect dietary information. Nevertheless, previous research has suggested an indirect effect of diet on hyperuricemia, the adjustment of BMI in the regression model would eliminate this indirect effect (10, 49), and a recent study also revealed that diet has a relatively minor role in determining serum urate levels (16). Third, other obesity measurements, such as waist circumference and hip circumference were not available, thus limited the comprehensive effect estimation of excess adiposity on hyperuricemia. Lastly, the generality of this study may be limited by its survey in specific areas of China.

## Conclusions

In summary, using representative samples from a general population in the core area, Beijing-Tianjin-Hebei, of China, our study revealed a high prevalence of hyperuricemia, and indicates that modifiable risk factors (overweight/obesity, alcohol consumption, cigarette smoking and sedentary behavior) contribute greatly to hyperuricemia cases based on their populational attributable fractions. Public health efforts including weight loss, reduction on alcohol consumption and cigarette smoking, and less sedentary time would be helpful for hyperuricemia prevention, especially in men.

## Data availability statement

The raw data supporting the conclusions of this article will be made available by reasonable request by sending email to the corresponding author (zhangminying@nankai.edu.cn).

## Ethics statement

The studies involving human participants were reviewed and approved by the Ethics Review Boards of Nankai University (NKUIRB2016063), Tianjin First Central Hospital (2017N052KY), Tianjin Union Medical Center (2018C02), and Hebei Medical University (2016021). The participants provided their written informed consent to participate in this study.

## Author contributions

Concept and data analysis: HH and MZ. Design: HH, MZ, and GS. Definition of intellectual content: PG, HH, JZ, SC, YN, FG, FL, RZ, QL, SM, BZ, and LP. Data acquisition: HH, MZ, PG, JH, JZ, SC, YN, FG, FL, RZ, QL, SM, and BZ. Statistical analysis and manuscript preparation: HH. Manuscript editing: MZ and GS. Manuscript review: PG, JH, JZ, SC, YN, FG, FL, RZ, QL, SM, BZ, and LP. MZ takes responsibility for the integrity of the work. All authors contributed to the article and approved the submitted version.

## Funding

This work was supported by the National Key R&D Program of China (No. 2016YFC0900600/2016YFC0900604), and the National Natural Science Foundation of China (82003531).

## Conflict of interest

The authors declare that the research was conducted in the absence of any commercial or financial relationships that could be construed as a potential conflict of interest.

## Publisher's note

All claims expressed in this article are solely those of the authors and do not necessarily represent those of their affiliated organizations, or those of the publisher, the editors and the reviewers. Any product that may be evaluated in this article, or claim that may be made by its manufacturer, is not guaranteed or endorsed by the publisher.

## References

[1] Fathallah-Shaykh SA Cramer MT. Uric acid and the kidney. Pediatr Nephrol. (2014) 29:999–1008. 10.1007/s00467-013-2549-x 23824181

[2] Feig DI Kang D Johnson RJ. Uric acid and cardiovascular risk. New Eng J Med. (2008) 359:1811–21. 10.1056/NEJMra0800885 18946066 PMC2684330

[3] Kuo CF Grainge MJ Zhang W Doherty M. Global epidemiology of gout: prevalence, incidence and risk factors. Nat Rev Rheumatol. (2015) 11:649–62. 10.1038/nrrheum.2015.91 26150127

[4] Kim Y Kang J Kim G. Prevalence of hyperuricemia and its associated factors in the general Korean population: an analysis of a population-based nationally representative sample. Clin Rheumatol. (2018) 37:2529–38. 10.1007/s10067-018-4130-2 29790110

[5] Bardin T Richette P. Impact of comorbidities on gout and hyperuricaemia: an update on prevalence and treatment options. BMC Med. (2017) 15:123. 10.1186/s12916-017-0890-9 28669352 PMC5494879

[6] Wu AH Gladden JD Ahmed M Ahmed A Filippatos G. Relation of serum uric acid to cardiovascular disease. Int J Cardiol. (2016) 213:4–7. 10.1016/j.ijcard.2015.08.110 26341316

[7] Otaki Y Konta T Ichikawa K Fujimoto S Iseki K Moriyama T. Possible burden of hyperuricaemia on mortality in a community-based population: a large-scale cohort study. Sci Rep. (2021) 11:8999. 10.1038/s41598-021-88631-8 33903733 PMC8076257

[8] He H Pan L Ren X Wang D Du J Cui Z. The effect of body weight and alcohol consumption on hyperuricemia and their attributable population fractions: a National health survey in China. Obes Facts. (2022) 15:216–27. 10.1159/000521163 34839297 PMC9021635

[9] He H Pan L Ren X Wang D Du J Cui Z. The effect of body adiposity and alcohol consumption on serum uric acid: a quantile regression analysis based on the China national health survey. Front Nutr. (2022) 8:724497. 10.3389/fnut.2021.724497 35111792 PMC8801605

[10] Choi HK McCormick N Lu N Rai SK Yokose C Zhang Y. Population Impact attributable to modifiable risk factors for hyperuricemia. Arthritis Rheumatol. (2019) 72:157–65. 10.1002/art.41067 31486212 PMC6935419

[11] Dong X Li Y Zhang L Liu X Tu R Wang Y. Independent and interactive effect of sitting time and physical activity on prevalence of hyperuricemia: the Henan Rural Cohort Study. Arthritis Rheumatol. (2021) 23:7. 10.1186/s13075-020-02385-8 33407821 PMC7789632

[12] Johnson RJ Bakris GL Borghi C Chonchol MB Feldman D Lanaspa MA. Hyperuricemia, acute and chronic kidney disease, hypertension, and cardiovascular disease: report of a scientific workshop organized by the national kidney foundation. Am J Kidney Dis. (2018) 71:851–65. 10.1053/j.ajkd.2017.12.009 29496260 PMC7286363

[13] Pan L Yang Z Wu Y Yin RX Liao Y Wang J. The prevalence, awareness, treatment and control of dyslipidemia among adults in China. Atherosclerosis. (2016) 248:2–9. 10.1016/j.atherosclerosis.2016.02.006 26978581

[14] Liu F Du G Song N Ma Y Li X Gao X. Hyperuricemia and its association with adiposity and dyslipidemia in Northwest China: results from cardiovascular risk survey in Xinjiang (CRS 2008–2012). Lipids Health Dis. (2020) 19:58. 10.1186/s12944-020-01211-z 32238146 PMC7115071

[15] Zapata-Diomedi B Barendregt JJ Veerman JL. Population attributable fraction: names, types and issues with incorrect interpretation of relative risks. Br J Sports Med. (2018) 52:212–3. 10.1136/bjsports-2015-095531 26964147

[16] Topless RKG Major TJ Florez JC Hirschhorn JN Cadzow M Dalbeth N. The comparative effect of exposure to various risk factors on the risk of hyperuricaemia: diet has a weak causal effect. Arthritis Res Ther. (2021) 23:75. 10.1186/s13075-021-02444-8 33663556 PMC7931603

[17] Zhang R Dong S Li Z. The economic and environmental effects of the Beijing-Tianjin-Hebei Collaborative Development Strategy— taking Hebei Province as an example. Environ Sci Pollut Res Int. (2020) 27:35692–702. 10.1007/s11356-020-09790-1 32601864 PMC7447681

[18] Li L Zhang Y Zeng C. Update on the epidemiology, genetics, and therapeutic options of hyperuricemia. Am J Transl Res. (2020) 12:3167–81. 32774692 PMC7407685

[19] Zhou BF. Predictive values of body mass index and waist circumference for risk factors of certain related diseases in Chinese adults–study on optimal cut-off points of body mass index and waist circumference in Chinese adults. Biomed Environ Sci. (2002) 15:83–96. 12046553

[20] Ma YC Zuo L Chen JH Luo Q Yu XQ Li Y. Modified glomerular filtration rate estimating equation for Chinese patients with chronic kidney disease. J Am Soc Nephrol. (2006) 17:2937–44. 10.1681/ASN.2006040368 16988059

[21] Goicoechea M de Vinuesa SG Verdalles U Ruiz-Caro C Ampuero J Rincon A. Effect of allopurinol in chronic kidney disease progression and cardiovascular risk. Clin J Am Soc Nephrol. (2010) 5:1388–93. 10.2215/CJN.01580210 20538833 PMC2924417

[22] Roalfe AK Holder RL Wilson S. Standardisation of rates using logistic regression: a comparison with the direct method. BMC Health Serv Res. (2008) 8:275. 10.1186/1472-6963-8-275 19113996 PMC2661894

[23] Rockhill B Newman B Weinberg C. Use and misuse of population attributable fractions. Am J Public Health. (1998) 88:15–9. 10.2105/AJPH.88.1.15 9584027 PMC1508384

[24] Zou G. A modified poisson regression approach to prospective studies with binary data. Am J Epidemiol. (2004) 159:702–6. 10.1093/aje/kwh090 15033648

[25] Song P Wang H Xia W Chang X Wang M An L. Prevalence and correlates of hyperuricemia in the middle-aged and older adults in China. Sci Rep. (2018) 8:4314. 10.1038/s41598-018-22570-9 29531237 PMC5847518

[26] Huang X Zhang W Tang W Liu Y Ning Y Huang C. Prevalence and associated factors of hyperuricemia among urban adults aged 35–79 years in southwestern China: a community-based cross-sectional study. Sci Rep. (2020) 10:15683. 10.1038/s41598-020-72780-3 32973308 PMC7515884

[27] Huang J Ma ZF Zhang Y Wan Z Li Y Zhou H. Geographical distribution of hyperuricemia in mainland China: a comprehensive systematic review and meta-analysis. Glob Health Res Policy. (2020) 5:52. 10.1186/s41256-020-00178-9 33292806 PMC7708223

[28] Qi D Liu J Wang C Wang L Zhang X Lin Q. Sex-specific differences in the prevalence of and risk factors for hyperuricemia among a low-income population in China: a cross-sectional study. Postgrad Med. (2020) 132:559–67. 10.1080/00325481.2020.1761133 32394762

[29] Hak AE Choi HK. Menopause, postmenopausal hormone use and serum uric acid levels in US women–the Third National Health and Nutrition Examination Survey. Arthritis Res Ther. (2008) 10:R116. 10.1186/ar2519 18822120 PMC2592803

[30] Benjamin EJ Muntner P Alonso A Bittencourt MS Callaway CW Carson AP. Heart disease and stroke statistics-2019 update: a report from the American Heart Association. Circulation. (2019) 139:e56–528. 10.1161/CIR.0000000000000659 30700139

[31] Joo JK Hong GP Han SE Lee YJ Kim SC Kim CW. The association between serum uric acid level and incidence of metabolic syndrome according to menopausal status in Korean women. J Menopausal Med. (2014) 20:126–32. 10.6118/jmm.2014.20.3.126 25580424 PMC4286657

[32] Stockl D Doring A Thorand B Heier M Belcredi P Meisinger C. Reproductive factors and serum uric acid levels in females from the general population: the KORA F4 study. PLoS ONE. (2012) 7:e32668. 10.1371/journal.pone.0032668 22427861 PMC3302793

[33] He H Pan L Liu F Ren X Cui Z Pa L. The mediation effect of body composition on the association between menopause and hyperuricemia: evidence from China National Health Survey. Front Endocrinol. (2022) 13:879384. 10.3389/fendo.2022.879384 35757401 PMC9226682

[34] Lin W Kao Y Lin H Li MS Luo T Fritz JM. Age difference in the combined effect of soda drinks consumption and body adiposity on hyperuricaemia in US adults. Public Health Nutr. (2021) 24:5756–68. 10.1017/S1368980021000513 33541468 PMC10195520

[35] Gao Y Jia S Li D Huang C Meng Z Wang Y. Prediction model of random forest for the risk of hyperuricemia in a Chinese basic health checkup test. Biosci Rep. (2021) 41:R20203859. 10.1042/BSR20203859 33749777 PMC8026814

[36] Kuwabara M Kuwabara R Niwa K Hisatome I Smits G Roncal-Jimenez C. Different risk for hypertension, diabetes, dyslipidemia, and hyperuricemia according to level of body mass index in Japanese and American subjects. Nutrients. (2018) 10:1011. 10.3390/nu10081011 30081468 PMC6115805

[37] Han Q Zhang D Zhao Y Liu L Li J Zhang F. Risk factors for hyperuricemia in Chinese centenarians and near-centenarians. Clin Interv Aging. (2019) 14:2239–47. 10.2147/CIA.S223048 31908434 PMC6927493

[38] He H Pan L Ren X Wang D Du J Cui Z. Joint effect of beer, spirits intake, and excess adiposity on hyperuricemia among chinese male adults: evidence from the China national health survey. Front Nutr. (2022) 9:806751. 10.3389/fnut.2022.806751 35273987 PMC8902589

[39] Nakamura K Sakurai M Miura K Morikawa Y Yoshita K Ishizaki M. Alcohol intake and the risk of hyperuricaemia: a 6-year prospective study in Japanese men. Nutr Metab Cardiovasc Dis. (2012) 22:989–96. 10.1016/j.numecd.2011.01.003 21421297

[40] Fanning N Merriman TR Dalbeth N Stamp LK. An association of smoking with serum urate and gout: a health paradox. Semin Arthritis Rheum. (2018) 47:825–42. 10.1016/j.semarthrit.2017.11.004 29398126

[41] Chen HG Sheng LT Wan ZZ Wang XC Lin YH Wang YX. The relationship between smoking and hyperuricemia in Chinese residents. Chin J Epidemiol. (2018) 52:524–9. 10.3760/cma.j.issn.0253-9624.2018.05.012 29747345

[42] Yang T Zhang Y Wei J Zeng C Li L Xie X. Relationship between cigarette smoking and hyperuricemia in middle-aged and elderly population: a cross-sectional study. Rheumatol Int. (2017) 37:131–6. 10.1007/s00296-016-3574-4 27704161

[43] Zhang S Wang Y Cheng J Huangfu N Zhao R Xu Z. Hyperuricemia and Cardiovascular Disease. Curr Pharm Des. (2019) 25:700–9. 10.2174/1381612825666190408122557 30961478

[44] Borghi C Agabiti-Rosei E Johnson RJ Kielstein JT Lurbe E Mancia G. Hyperuricaemia and gout in cardiovascular, metabolic and kidney disease. Eur J Intern Med. (2020) 80:1–11. 10.1016/j.ejim.2020.07.006 32739239

[45] Chen J Wen CP Wu SB Lan J Tsai MK Tai Y. Attenuating the mortality risk of high serum uric acid: the role of physical activity underused. Ann Rheum Dis. (2015) 74:2034–42. 10.1136/annrheumdis-2014-205312 25053714

[46] Park DY Kim YS Ryu SH Jin YS. The association between sedentary behavior, physical activity and hyperuricemia. Vasc Health Risk Manag. (2019) 15:291–9. 10.2147/VHRM.S200278 31616149 PMC6698593

[47] Bluher M. Obesity: global epidemiology and pathogenesis. Nat Rev Endocrinol. (2019) 15:288–98. 10.1038/s41574-019-0176-8 30814686

[48] Hong R Huang J Xu C Zhang X Mi F Xu F. Association of sedentary behavior and physical activity with hyperuricemia and sex differences: results from the China multi-ethnic cohort study. J Rheumatol. (2022) 49:513–22. 10.3899/jrheum.211180 35169050

[49] Williams PT. Effects of diet, physical activity and performance, and body weight on incident gout in ostensibly healthy, vigorously active men. Am J Clin Nutr. (2008) 87:1480–7. 10.1093/ajcn/87.5.1480 18469274 PMC4090353

