# Supplementary material for: Prevalence of hyperuricemia and the population attributable fraction of modifiable risk factors: Evidence from a general population cohort in China
Source: Front Public Health. 2022 Jul 28;10:936717. doi: 10.3389/fpubh.2022.936717 (PMC9366258; doi:10.3389/fpubh.2022.936717)
Supplement: Supplementary file 1 [file Table_1.DOCX]

**Table S1**. The modified effect of body mass index on the influence of modifiable risk factors on hyperuricemia

| **Modifiable risk factors** | % of exposure | PAF (%) | 95%CI or PAF | | P |
| --- | --- | --- | --- | --- | --- |
| **Under/normal weight** |  |  |  | |  |
| *Current drinking* | 38.15 | 10.83 | 7.54 | 14.13 | <0.001 |
| *Ever smoking* | 40.02 | 6.21 | 2.07 | 10.35 | 0.003 |
| *Daily sedentary time* |  |  |  |  |  |
| 2-4h | 19.94 | 5.75 | 2.35 | 9.15 | 0.001 |
| 4-6h | 29.48 | 9.42 | 4.77 | 14.07 | <0.001 |
| >6h | 38.13 | 10.46 | 4.05 | 16.88 | 0.001 |
| **Overweight/obesity** |  |  |  |  |  |
| *Current drinking* | 42.31 | 2.64 | 0.31 | 5.97 | 0.026 |
| *Ever smoking* | 42.66 | 2.61 | 0.24 | 4.98 | 0.031 |
| *Daily sedentary time* |  |  |  |  |  |
| 2-4h | 20.61 | 2.28 | 0.16 | 4.39 | 0.035 |
| 4-6h | 29.49 | 5.63 | 3.01 | 8.26 | <0.001 |
| >6h | 39.79 | 7.16 | 3.57 | 10.75 | <0.001 |

PAF: population attributable fraction; CI: confidence interval. The PAF was adjusted for age, geographic areas and serum creatinine

**Table S2.** Simulated prevalence of hyperuricemia given changed PAF of MRFs, stratified by sex.

|  | **Male** | | | |  | **Female** | | | | |
| --- | --- | --- | --- | --- | --- | --- | --- | --- | --- | --- |
| Overweight/obesity  prevalence | PAF (%) | 95% CI | | Hyperuricemia prevalence |  | Overweight/obesity  prevalence | PAF (%) | 95% CI | | Hyperuricemia prevalence |
| 80 | 35.61 | 32.71 | 38.52 | 29.41 |  | 80 | 44.57 | 40.86 | 48.28 | 13.21 |
| 75 | 33.39 | 30.66 | 36.11 | 28.79 |  | 75 | 41.78 | 38.30 | 45.27 | 12.92 |
| 70 | 31.16 | 28.62 | 33.70 | 28.18 |  | 70 | 39 | 35.75 | 42.25 | 12.62 |
| **66.26*** | 29.50 | 27.09 | 31.90 | 27.72 |  | 65 | 36.21 | 33.20 | 39.23 | 12.32 |
| 60 | 26.71 | 24.53 | 28.89 | 26.94 |  | 60 | 33.43 | 30.64 | 36.21 | 12.02 |
| 55 | 24.48 | 22.49 | 26.48 | 26.32 |  | 55 | 30.64 | 28.09 | 33.19 | 11.73 |
| 50 | 22.25 | 20.44 | 24.07 | 25.71 |  | 50 | 27.86 | 25.53 | 30.18 | 11.43 |
| 45 | 20.03 | 18.40 | 21.67 | 25.09 |  | 45 | 25.07 | 22.98 | 27.16 | 11.13 |
| 40 | 17.81 | 16.35 | 19.26 | 24.48 |  | **37.65*** | 20.98 | 19.23 | 22.72 | 10.69 |
| 35 | 15.58 | 14.31 | 16.85 | 23.86 |  | 35 | 19.50 | 17.87 | 21.12 | 10.53 |
| 30 | 13.35 | 12.27 | 14.44 | 23.24 |  | 30 | 16.71 | 15.32 | 18.11 | 10.24 |
| 25 | 11.33 | 10.22 | 12.04 | 22.68 |  | 25 | 13.93 | 12.77 | 15.09 | 9.94 |
| 20 | 8.90 | 8.18 | 9.63 | 22.01 |  | 20 | 11.14 | 10.21 | 12.07 | 9.64 |
| 15 | 6.68 | 6.13 | 7.22 | 21.39 |  | 15 | 8.36 | 7.66 | 9.05 | 9.34 |

*Current exposure prevalence. PAF: population attributable fraction; MRF: modifiable risk factor; CI: confidence interval.

**Table S3.** The population attributable risk of modifiable risk factors for hyperuricemia in women, stratified by menopausal status.

| **Modifiable risk factors** | % of exposure | PAF (%) | 95%CI or PAF | | P |
| --- | --- | --- | --- | --- | --- |
| **Pre-menopausal** |  |  |  | |  |
| *Overweight* | 23.29 | 12.36 | 10.64 | 14.08 | <0.001 |
| *Obesity* | 7.39 | 5.51 | 5.16 | 5.86 | <0.001 |
| *Current drinking* | 5.17 | -1.19 | -3.12 | 0.75 | 0.230 |
| *Ever smoking* | 1.12 | 0.34 | -0.01 | 0.68 | 0.055 |
| *Daily sedentary time* |  |  |  |  |  |
| 2-4h | 14.39 | 1.16 | -3.18 | 5.50 | 0.601 |
| 4-6h | 27.34 | 1.98 | -5.69 | 9.65 | 0.256 |
| >6h | 51.17 | 10.62 | -1.03 | 22.26 | 0.074 |
| **Post-menopausal** |  |  |  |  |  |
| *Overweight* | 39.27 | 16.63 | 12.86 | 20.40 | <0.001 |
| *Obesity* | 13.07 | 7.48 | 6.36 | 8.60 | <0.001 |
| *Current drinking* | 3.12 | -0.70 | -2.41 | 1.02 | 0.426 |
| *Ever smoking* | 1.85 | 0.81 | 0.38 | 1.24 | <0.001 |
| *Daily sedentary time* |  |  |  |  |  |
| 2-4h | 24.49 | 1.36 | -4.37 | 7.08 | 0.643 |
| 4-6h | 29.41 | 1.73 | -5.15 | 8.61 | 0.623 |
| >6h | 34.46 | -0.11 | -8.89 | 8.68 | 0.981 |

PAF: population attributable fraction; CI: confidence interval. The covariates adjusted in the regression model were age, geographic areas, body mass index, alcohol consumption, smoking status and daily sedentary time.

**Table S4.** The population attributable risk of modifiable risk factors for hyperuricemia excluding patients with diagnosed chronic diseases, stratified by sex.

| **Modifiable risk factors** | % of exposure | PAF (%) | 95%CI or PAF | | P |
| --- | --- | --- | --- | --- | --- |
| **Male** |  |  |  | |  |
| *Overweight* | 43.64 | 17.19 | 14.87 | 19.51 | <0.001 |
| *Obesity* | 15.59 | 8.70 | 8.04 | 9.36 | <0.001 |
| *Current drinking* | 39.74 | 4.03 | 1.45 | 6.62 | 0.002 |
| *Ever smoking* | 38.96 | 2.62 | -0.30 | 5.27 | 0.052 |
| *Daily sedentary time* |  |  |  |  |  |
| 2-4h | 19.26 | 2.77 | 0.32 | 5.22 | 0.027 |
| 4-6h | 29.69 | 6.85 | 3.63 | 10.06 | <0.001 |
| >6h | 41.36 | 8.41 | 3.81 | 13.02 | <0.001 |
| **Female** |  |  |  |  |  |
| *Overweight* | 24.46 | 12.44 | 10.73 | 14.15 | <0.001 |
| *Obesity* | 5.85 | 4.20 | 3.90 | 4.51 | <0.001 |
| *Current drinking* | 5.22 | -1.37 | -3.48 | 0.74 | 0.203 |
| *Ever smoking* | 1.81 | 0.82 | 0.39 | 1.24 | 0.002 |
| *Daily sedentary time* |  |  |  |  |  |
| 2-4h | 15.86 | -0.92 | -5.63 | 3.79 | 0.701 |
| 4-6h | 28.22 | 0.32 | -6.97 | 7.60 | 0.932 |
| >6h | 48.01 | 6.26 | -4.19 | 16.72 | 0.240 |

PAF: population attributable fraction; CI: confidence interval. The covariates adjusted in the regression model were age, geographic areas, body mass index, alcohol consumption, smoking status, daily sedentary time, and serum creatinine. Menopausal status was additionally adjusted in women.

**Table S5.** The population attributable risk of modifiable risk factors for hyperuricemia, stratified by age and sex.

| **Modifiable risk factors** | % of exposure | PAF (%) | 95% CI or PAF | | P |
| --- | --- | --- | --- | --- | --- |
| **Male**  **Aged 18-29** |  |  |  | |  |
| *Overweight/obesity* | 55.08 | 26.23 | 22.08 | 30.38 | <0.001 |
| *Current drinking* | 35.45 | -1.29 | -6.07 | 3.49 | 0.598 |
| *Ever smoking* | 33.66 | 0.19 | -4.31 | 4.70 | 0.932 |
| *Daily sedentary time* |  |  |  |  |  |
| 2-4h | 19.79 | -1.27 | -6.38 | 3.84 | 0.627 |
| 4-6h | 27.45 | 2.43 | -3.29 | 8.16 | 0.404 |
| >6h | 44.08 | 0.82 | -8.87 | 10.51 | 0.869 |
| **Aged 30-39** |  |  |  |  |  |
| *Overweight/obesity* | 66.82 | 33.80 | 30.01 | 37.60 | <0.001 |
| *Current drinking* | 40.68 | -4.77 | -8.72 | 0.00 | 0.018 |
| *Ever smoking* | 35.15 | 3.79 | 1.04 | 6.53 | 0.007 |
| *Daily sedentary time* |  |  |  |  |  |
| 2-4h | 16.99 | -0.68 | -4.06 | 270 | 0.693 |
| 4-6h | 31.19 | 3.82 | -1.05 | 8.68 | 0.124 |
| >6h | 45.12 | 3.66 | -3.63 | 10.96 | 0.325 |
| **Aged 40-49** |  |  |  |  |  |
| *Overweight/obesity* | 68.15 | 28.47 | 22.75 | 34.18 | <0.001 |
| *Current drinking* | 45.38 | -5.47 | -11.32 | 0.38 | 0.067 |
| *Ever smoking* | 42.87 | 4.16 | -0.29 | 8.62 | 0.067 |
| *Daily sedentary time* |  |  |  |  |  |
| 2-4h | 19.33 | 5.12 | 1.92 | 8.32 | 0.002 |
| 4-6h | 30.73 | 7.31 | 2.23 | 12.39 | 0.005 |
| >6h | 38.85 | 9.45 | 1.67 | 17.24 | 0.017 |
| **Aged 50-59** |  |  |  |  |  |
| *Overweight/obesity* | 68.95 | 22.81 | 15.47 | 30.14 | <0.001 |
| *Current drinking* | 46.21 | -13.26 | -21.32 | -5.19 | 0.001 |
| *Ever smoking* | 51.65 | 1.44 | -5.23 | 8.10 | 0.672 |
| *Daily sedentary time* |  |  |  |  |  |
| 2-4h | 19.80 | 6.22 | 2.83 | 9.60 | <0.001 |
| 4-6h | 27.92 | 10.07 | 5.76 | 14.38 | <0.001 |
| >6h | 36.25 | 14.59 | 9.35 | 19.83 |  |
| **Aged 60-80** |  |  |  |  |  |
| *Overweight/obesity* | 68.46 | 23.42 | 14.30 | 32.54 | <0.001 |
| *Current drinking* | 33.66 | -2.74 | -9.13 | 3.66 | 0.402 |
| *Ever smoking* | 44.95 | 5.38 | -1.44 | 12.21 | 0.122 |
| *Daily sedentary time* |  |  |  |  |  |
| 2-4h | 27.82 | 6.35 | 0.05 | 12.65 | 0.048 |
| 4-6h | 29.37 | 7.76 | 1.46 | 14.06 | 0.016 |
| >6h | 30.16 | 7.38 | 0.43 | 14.33 | 0.037 |
| **Female** |  |  |  |  |  |
| *Overweight/obesity* |  |  |  |  |  |
| Aged 18-29 | 17.47 | 11.21 | 9.53 | 12.88 | <0.001 |
| Aged 30-39 | 28.49 | 19.46 | 17.66 | 21.27 | <0.001 |
| Aged 40-49 | 41.17 | 22.32 | 17.66 | 26.98 | <0.001 |
| Aged 50-59 | 48.70 | 24.03 | 18.66 | 29.41 | <0.001 |
| Aged 60-80 | 55.67 | 23.24 | 16.69 | 29.80 | <0.001 |

PAF: population attributable fraction; CI: confidence interval. The covariates adjusted in the regression model were geographic areas, body mass index, alcohol consumption, smoking status, daily sedentary time, and serum creatinine. Menopausal status was additionally adjusted in women aged over 40.
